# Human adult and adolescent biodistribution and dosimetry of the synaptic vesicle glycoprotein 2A radioligand ^11^C-UCB-J

**DOI:** 10.1186/s13550-020-00670-w

**Published:** 2020-07-14

**Authors:** Jason Bini, Daniel Holden, Kathryn Fontaine, Tim Mulnix, Yihuan Lu, David Matuskey, Jim Ropchan, Nabeel Nabulsi, Yiyun Huang, Richard E. Carson

**Affiliations:** Yale PET Center, Department of Radiology and Biomedical Imaging, Yale University School of Medicine, 801 Howard Avenue, PO Box 208048, New Haven, CT USA

**Keywords:** Dosimetry, Carbon-11, SV2A, UCB-J, Adolescent

## Abstract

**Abstract:**

The ability to quantify synaptic density in vivo in human adults and adolescents is of vital importance to understanding neuropsychiatric disorders. Here, we performed whole-body scans to determine organ radiation dosimetry of ^11^C-UCB-J in humans.

**Methods:**

Dynamic whole-body PET scans were performed in four healthy adults after injection of ^11^C-UCB-J. Regions of interest (ROIs) were drawn manually for the brain, heart, stomach, kidneys, liver, pancreas, spleen, gallbladder, lungs, urinary bladder, and intestines. ROIs were applied to dynamic images to generate time-activity curves (TACs). Decay correction was removed from TACs, and the area under the curve (AUC) for each ROI was calculated. AUCs were then normalized by injected activity and organ volumes to produce radioligand residence times for each organ. These times were then used as input into the OLINDA/EXM 1.0 software to determine the total radiation dose in each organ and the effective dose for these OLINDA models: 55-kg female, 70-kg male, and 15-year-old adolescent.

**Results:**

Visual evaluation detected high uptake in the liver, brain, gallbladder, gastrointestinal tract, and urinary bladder. The dose-limiting organ was the urinary bladder for adult males (0.0224 mSv/MBq) and liver for adult females (0.0248 mSv/MBq) with single-study dose limits of 2239 MBq and 2017 MBq ^11^C-UCB-J, respectively. For adolescents, the large intestine was the dose-limiting organ (0.0266 mSv/MBq) with a single-study dose limit of 188 MBq.

**Conclusions:**

^11^C-UCB-J dosimetry in adults is consistent with those for many carbon-11-labeled ligands. Overall, ^11^C-UCB-J can be used safely in adolescents, as in adults, to measure synaptic density in various neuropsychiatric and other relevant disorders.

## Introduction

Many neuropsychiatric disorders are associated with alterations in synaptic density and synaptic pruning. The ability to quantify synaptic density in vivo in human adults and adolescents is of vital importance to understanding the etiology of these diseases and associated changes in synaptic density. The synaptic vesicle glycoprotein 2A (SV2A) has recently been proposed as a marker of synaptic density that can be measured in vivo using several positron emission tomography (PET) radioligands, ^11^C-UCB-H [1], ^11^C-UCB-A [2], and ^11^C-UCB-J [3]. ^11^C-UCB-J has been evaluated in baboons [3], rhesus monkeys [4], and human studies [3, 5] and demonstrated excellent kinetic properties as a PET radioligand [5]. ^11^C-UCB-J has also been used to examine synaptic density in Alzheimer’s disease [6], depression [7], epilepsy [3], Parkinson’s disease [8], and schizophrenia [9]. Our group has previously demonstrated that SV2A is an alternate synaptic marker to synaptophysin [3], suggesting ^11^C-UCB-J could be used to assess synaptic density in vivo for many psychiatric disorders. This tracer may also be of high value for the study of autism spectrum disorder (ASD), Huntington’s disease, multiple sclerosis, and stroke [10].

In several neurodevelopmental disorders that present in children or adolescents (e.g., schizophrenia and ASD), synaptic pruning may be disrupted, highlighting the need for an in vivo tool to understand such changes longitudinally [11, 12]. Many ASD risk genes are associated with changes in synaptic plasticity [13], suggesting that ^11^C-UCB-J may provide a valuable in vivo tool for investigating ASD in adolescents. The ability to scan adolescents, as well as adults, to gain further understanding in vivo of disease progression possibly related to synaptic pruning is an important potential application of this tracer. In order to facilitate research imaging studies, especially in adolescents, radiation doses must be monitored to not exceed single scan and yearly limits set forth in the US FDA code of federal regulation: Section 21 CFR 361.1(b)(3)(ii) while maintaining suitable image quality.

In the current study, we performed dynamic whole-body scans with ^11^C-UCB-J in adults for organ dosimetry calculations based on adult and adolescent phantoms to provide dose estimates for both populations. We also compared residence times in humans to previously acquired non-human primate (NHP) data to examine species differences in each organ [4].

## Methods

### Subjects

Four healthy individuals (2F/2M, 26–47 years) underwent PET/CT imaging after injection of ^11^C-UCB-J. Mean injected activity was 292 ± 151 MBq, mean injected mass was 0.016 ± 0.014 μg/kg, and mean molar activity at the time of injection was 93 ± 29 MBq/nmol. The maximum injected mass of UCB-J in the current study was 0.04 μg/kg (2.0 μg). Subject demographics are presented in Table 1. The study was approved by the Yale University Human Investigation Committee and Radiation Safety Committees, and all subjects signed a written informed consent. Previously, dosimetry studies were performed in rhesus monkeys (*Macaca mulatta*) (*n* = 2 males and 2 females) according to a protocol approved by the Yale University Institutional Animal Care and Use Committee and detailed methods have been published [4]. Briefly, for the NHP dosimetry scans, the mean injected radioactivity was 170 ± 15 MBq, with a molar activity of 371 ± 42 MBq/nmol at the time of injection and an injected mass dose of 0.02 ± 0.01 μg/kg (*n* = 4). Whole-body dynamic PET scans were performed on the Siemens mCT-X (13 passes; 1 × 1.5 min, 1 × 2 min, 3 × 3 min, 3 × 6 min, 3 × 15 min, 2 × 25 min).

**Table 1:** Demographic information, injected activity dose, and injected mass for each subject (*n* = 4)

Individual	Sex	Age (years)	Height (m)	Weight (kg)	Injected activity (MBq)	Injected mass (μg/kg)
**1**	F	40	1.60	77.0	335	0.010
**2**	F	26	1.62	55.0	484	0.037
**3**	M	47	1.78	90.5	210	0.014
**4**	M	44	1.80	85.7	140	0.004
**Mean ± SD**		39.3 ± 9.3	1.7 ± 0.1	77.1 ± 15.7	292 ± 151	0.016 ± 0.014

### PET/CT imaging

All subjects underwent PET/CT imaging on the Siemens Biograph mCT-X PET/CT system (Siemens Healthcare). Dynamic PET scans were acquired with continuous bed motion from the mid-thigh to the top of the head (29 passes; 4 × 60 s, 2 × 120 s, 23 × 5 min). Bed speed was altered to achieve similar pass acquisition times to account for height differences between subjects. Dynamic images were reconstructed using an ordered subset expectation maximization-based algorithm including point spread function and time-of-flight information. Attenuation correction was performed using the CT acquisition. Regions of interest (ROIs) were drawn manually for the brain, heart, stomach, kidneys, liver, pancreas, and spleen on a summed 0–10-min PET image. ROIs for the lungs, urinary bladder, and intestines were drawn on a summed 0–30-min PET image. Since the gallbladder size varies during the scan, the gallbladder ROI was defined to include its maximum extent. All ROIs were applied to the dynamic PET images to generate time-activity curves (TACs).

### Dosimetry

Decay correction was removed from the TACs, and the area under the curve (AUC) was calculated for each organ. The trapezoidal rule was used to estimate the AUC from dynamic PET data. Extrapolation was performed by using the decay constant for ^11^C for all TACs from the midpoint of the last dynamic frame to infinity to determine AUC beyond the end of the scan.

AUC measures were then normalized by the injected activity and organ volume estimates from either the 55-kg adult female/15-year-old adolescent or 70-kg adult male phantom to produce residence times in hours for each organ [14, 15]. For the gallbladder, the normalization used the organ volume measured from the gallbladder ROIs, as was done previously [16]. The residence time in the remainder of the body was calculated as the maximum possible residence time based on ^11^C decay minus the sum of residence times of all defined ROIs. We also compared organ residence times in humans to those previously acquired in non-human primates to examine potential species differences [4].

Residence times for each organ were then used as input into the OLINDA/EXM 1.0 software to determine the total radiation dose in each organ and effective dose (mSv/MBq) for each standard OLINDA model: 55-kg female, 70-kg male, and 15-year-old adolescent [15, 17, 18]. The 15-year-old adolescent model does not have separate male and female versions; therefore, residence times for all four individuals were input into the OLINDA 15-year-old adolescent model and the total dose to each organ was averaged over the four individuals to determine organ dosimetry for adolescents [14].

## Results

**Fig. 1:**
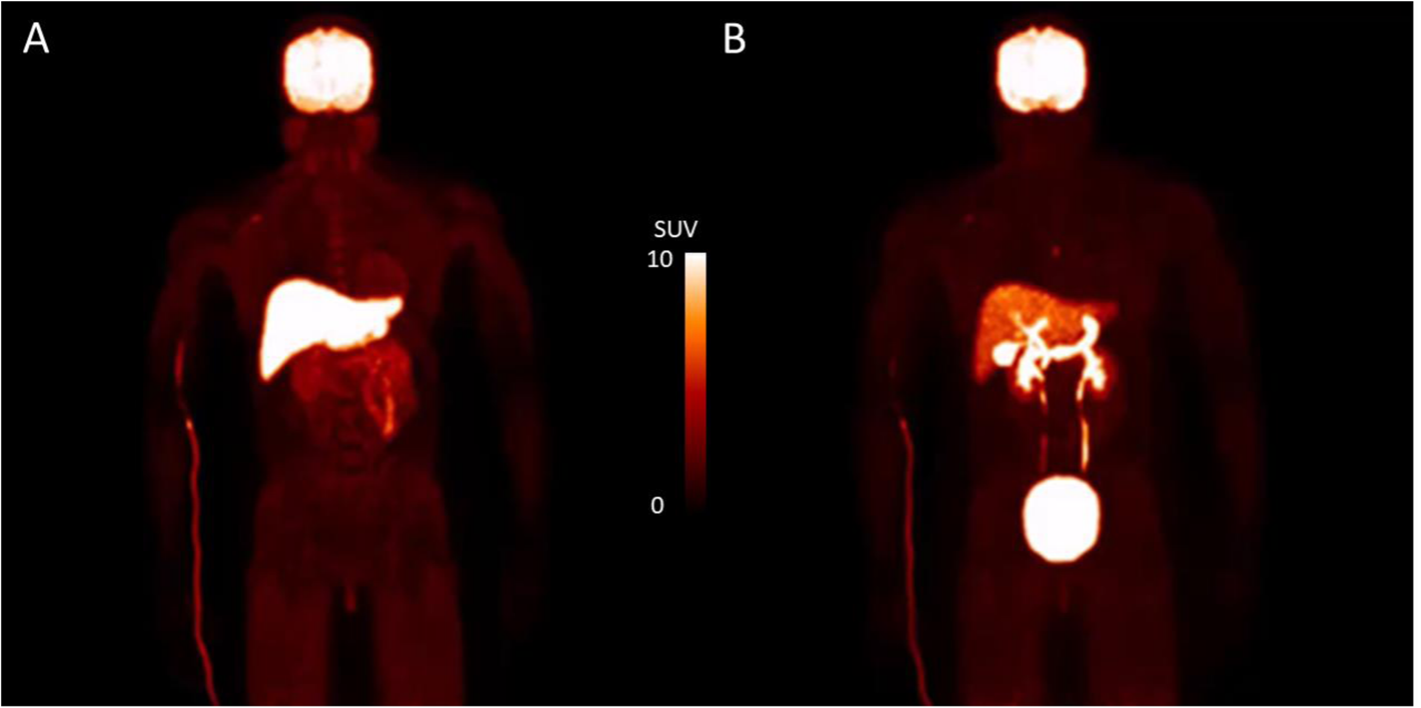
Representative maximum intensity projection images of ^11^C-UCB-J: **a** 2–10 min, **b** 30–60 min. SUV scale, 0–10

**Fig. 2:**
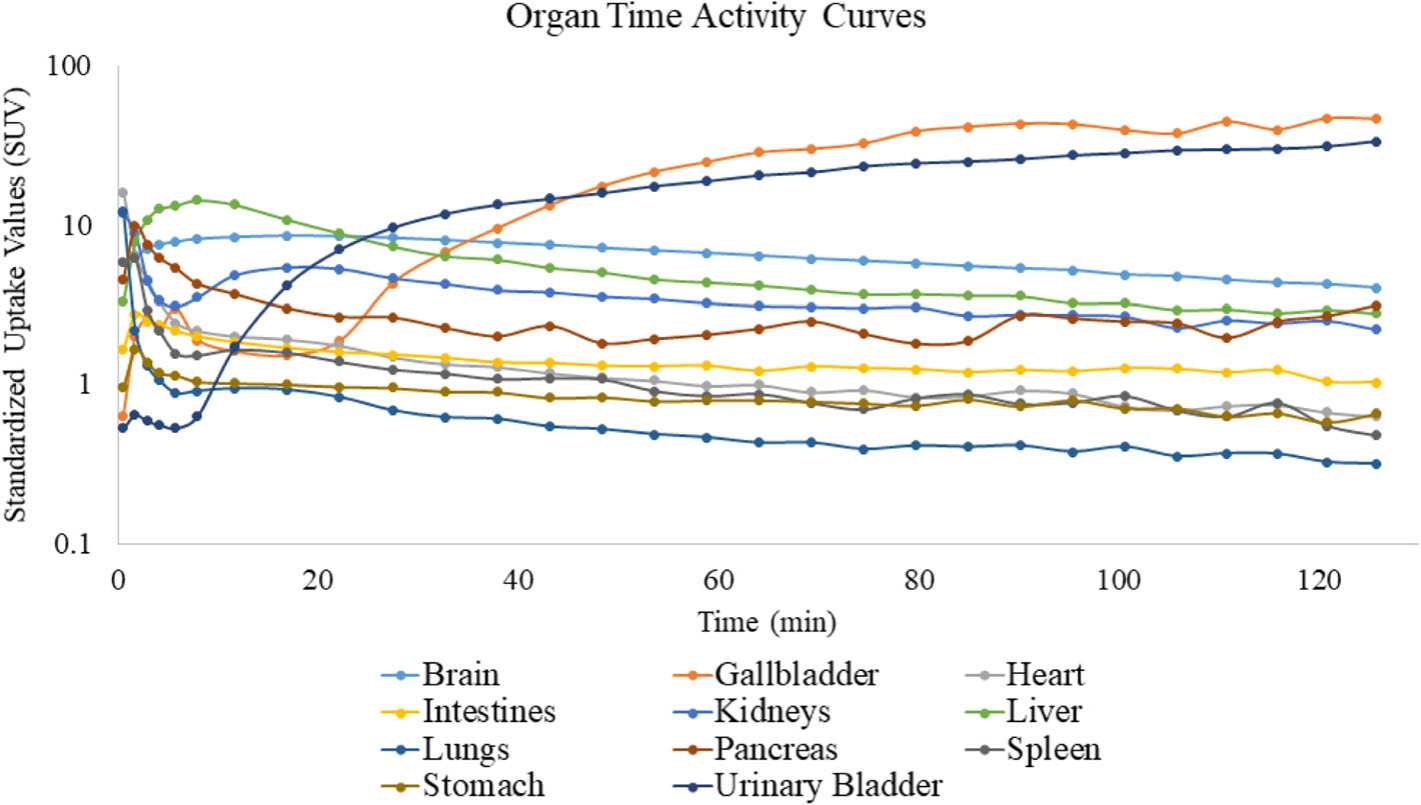
Representative decay-corrected time-activity curves of ^11^C-UCB-J for organs used in dosimetry calculations, in standardized uptake value (log scale)

Visual inspection of the dosimetry scans detected very high early uptake in the liver and brain (2–10 min) (Fig. 1a). Uptake at 30–60 min was clearly visible in the gallbladder, gastrointestinal tract, and urinary bladder, with high radioactivity levels remaining in the brain and liver (Fig. 1b). Representative decay-corrected standardized uptake values (SUV) TACs of ^11^C-UCB-J for organs used in dosimetry calculations demonstrated that urinary bladder activity increased continually until the end of the acquisition. While the gallbladder has ejected its contents, up to three times, its SUV values were still the highest at the end of the scan (Fig. 2).

Residence times for the five highest dose-limiting organs for both sexes are summarized in Table 2. Higher residence times were seen for females in the brain, gallbladder, liver, and lower intestines, and for males in the bladder with a higher remainder.

**Table 2:** Residence times of ^11^C-UCB-J in the human brain, gallbladder, lower intestine, liver, lower intestine, urinary bladder, and remainder of the body (hours). All values displayed as mean ± standard deviation

Organ	Females (***n*** = 2)	Males (***n*** = 2)	All (***n*** = 4)
**Brain**	0.0872 ± 0.0008	0.0595 ± 0.0036	0.0733 ± 0.0161
**Gallbladder**	0.0036 ± 0.0009	0.0029 ± 0.0017	0.0033 ± 0.0012
**Liver**	0.1078 ± 0.0062	0.0834 ± 0.0045	0.0965 ± 0.0148
**Lower intestine**	0.0223 ± 0.0086	0.0209 ± 0.0007	0.0216 ± 0.0051
**Urinary bladder**	0.0215 ± 0.0099	0.0303 ± 0.0106	0.0259 ± 0.0010
**Remainder**	0.2071 ± 0.0201	0.2532 ± 0.0201	0.2302 ± 0.0313

**Fig. 3:**
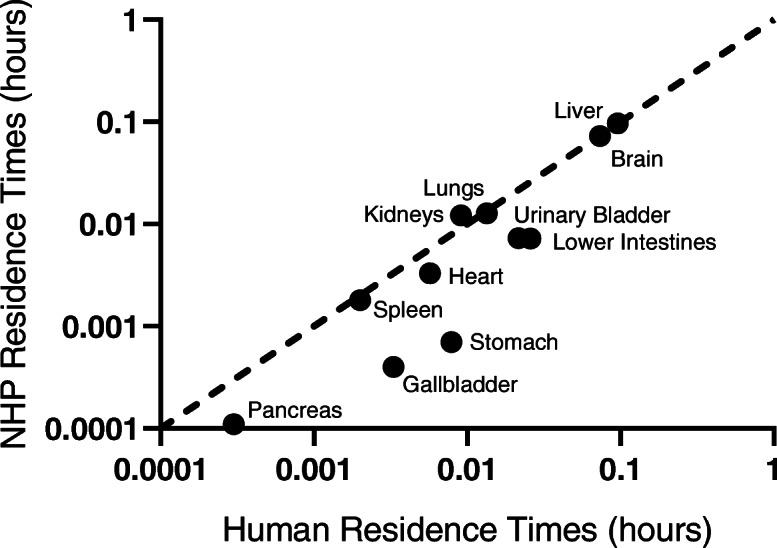
Comparison of residence times for 11 organs of humans and rhesus monkey (*Macaca mulatta*) non-human primates (NHP), on a log-log plot

Organ residence times for human and NHP were plotted for all ROIs (Fig. 3), and the values are summarized in Table 3. The organs with the highest residence times (brain and liver) matched extremely well; however, between human and NHP, the digestive organs (stomach, large intestine, pancreas, and gallbladder) and the urinary bladder in NHP had 66–91% shorter residence times than humans, likely due to slowing of the digestive tract by anesthesia and related drugs administered when conducting NHP scans.

**Table 3:** Organ residence times (hours) of ^11^C-UCB-J in humans in comparison with those in non-human primate (NHP). All values displayed as mean ± standard deviation

Organ	Mean ALL human (***n*** = 4)	Mean ALL NHP (***n*** = 4)	Percent difference (%)
**Brain**	0.0733 ± 0.0161	0.0729 ± 0.0045	**− 1**
**Gallbladder**	0.0033 ± 0.0012	0.0004 ± 0.0002	**− 88**
**Heart**	0.0057 ± 0.0009	0.0033 ± 0.0013	**− 42**
**Lower intestine**	0.0216 ± 0.0051	0.0073 ± 0.0020	**− 66**
**Kidneys**	0.0091 ± 0.0008	0.0122 ± 0.0023	**34**
**Liver**	0.0956 ± 0.0148	0.0965 ± 0.0407	**1**
**Lungs**	0.0134 ± 0.0063	0.0127 ± 0.0061	**− 5**
**Pancreas**	0.0003 ± 0.0001	0.0001 ± 0.0001	**− 66**
**Spleen**	0.0020 ± 0.0002	0.0018 ± 0.0005	**− 10**
**Stomach**	0.0079 ± 0.0067	0.0007 ± 0.0002	**− 91**
**Urinary bladder**	0.0259 ± 0.0010	0.0072 ± 0.0039	**− 72**
**Remainder**	0.2302 ± 0.0313	0.2730 ± 0.0455	**19**

Mean total absorbed doses in target organs derived from the 55-kg adult female, 70-kg adult male, and 15-year-old phantoms are shown in Table 4. The dose-limiting organ was the urinary bladder for adult males (0.0224 mSv/MBq) and liver for females (0.0248 mSv/MBq) with single-study dose limits of 2239 MBq (60.5 mCi) and 2017 MBq (54.5 mCi), respectively. The dose-limiting organ in adolescents was the lower intestines (0.0266 mSv/MBq). Based on federal regulation Section 21 CFR 361.1(b)(3)(ii), adult single scan and yearly organ dose limits are 5.0 rem and 15.0 rem, respectively. This federal regulation recommends 1/10th of the adult research dose for a research subject under 18 years of age. Based on these regulations, the single-study radiation dose limit for pediatric scans is 188 MBq (5.1 mCi) for adolescents. The mean effective dose (ED) for adults was 7.6 μSv/MBq, while the adolescent mean ED was 8.8 μSv/MBq.

**Table 4:** Mean organ radiation doses (mSv/MBq). Adult male and female values were based on the residence times measured in males and females, respectively (*n* = 2, each). For the adolescent calculation, the 4 residence time values were used, with the exception of reproductive organs

	55-kg female		70-kg male		15-year-old adolescents	
	Mean	+/−	Mean	+/−	Mean	+/−
Adrenals	3.71E**−**03	9.19E**−**05	2.96E**−**03	8.49E**−**05	3.63E**−**03	9.57E**−**05
**Brain**	**2.31E−02**	**2.83E−04**	**1.38E−02**	**7.78E−04**	**1.87E−02**	**1.75E−03**
Breasts	1.86E**−**03	1.84E**−**04	1.63E**−**03	9.90E**−**05	1.94E**−**03	1.52E**−**04
**Gallbladder wall**	**1.40E−02**	**2.33E−03**	**1.03E−02**	**3.66E−03**	**1.39E−02**	**3.37E−03**
**LLI wall**	**2.34E−02**	**8.13E−03**	**2.06E−02**	**6.36E−04**	**2.66E−02**	**5.84E−03**
Small intestine	2.95E**−**03	1.77E**−**04	2.71E**−**03	1.41E**−**05	3.22E**−**03	1.75E**−**04
Stomach wall	6.71E**−**03	4.36E**−**03	6.95E**−**03	4.46E**−**03	7.88E**−**03	4.38E**−**03
ULI wall	3.03E**−**03	8.49E**−**05	2.62E**−**03	2.12E**−**05	3.05E**−**03	1.21E**−**04
Heart wall	4.97E**−**03	6.15E**−**04	3.91E**−**03	1.20E**−**04	5.12E**−**03	4.70E**−**04
Kidneys	1.07E**−**02	3.54E**−**04	9.56E**−**03	1.05E**−**03	1.18E**−**02	8.38E**−**04
**Liver**	**2.48E−02**	**1.27E−03**	**1.48E−02**	**7.78E−04**	**2.17E−02**	**3.87E−03**
Lungs	6.20E**−**03	3.34E**−**03	4.27E**−**03	4.81E**−**04	6.23E**−**03	2.55E**−**03
Muscle	2.21E**−**03	6.36E**−**05	2.00E**−**03	6.36E**−**05	2.33E**−**03	1.38E**−**04
Ovaries	3.46E**−**03	4.24E**−**04			3.53E**−**03	4.17E**−**04
Pancreas	3.91E**−**03	4.67E**−**04	2.83E**−**03	2.97E**−**04	4.12E**−**03	4.23E**−**04
Red marrow	2.39E**−**03	4.95E**−**05	2.05E**−**03	5.66E**−**05	2.46E**−**03	9.25E**−**05
Osteogenic cells	3.40E**−**03	1.98E**−**04	2.81E**−**03	1.41E**−**04	3.47E**−**03	2.46E**−**04
Skin	1.72E**−**03	9.90E**−**05	1.55E**−**03	7.78E**−**05	1.80E**−**03	1.28E**−**04
Spleen	4.69E**−**03	6.36E**−**05	4.50E**−**03	1.70E**−**04	5.50E**−**03	1.81E**−**04
Testes			1.92E**−**03	1.41E**−**05	2.40E**−**03	1.41E**−**05
Thymus	2.19E**−**03	2.55E**−**04	1.92E**−**03	1.27E**−**04	2.31E**−**03	2.02E**−**04
Thyroid	1.84E**−**03	1.84E**−**04	1.89E**−**03	1.13E**−**04	2.25E**−**03	1.93E**−**04
**Urinary bladder wall**	**2.18E−02**	**9.19E−03**	**2.24E−02**	**7.00E−03**	**2.43E−02**	**8.13E−03**
Uterus	3.18E**−**03	3.46E**−**04			3.23E**−**03	3.39E**−**04
Total body	3.48E**−**03	4.95E**−**05	2.75E**−**03	4.95E**−**05	3.47E**−**03	5.56E**−**05
Effective dose (mSv/MBq)	8.20E**−**03	1.69E**−**03	6.97E**−**03	8.70E**−**04	8.79E**−**03	1.25E**−**03

## Discussion

We have performed dosimetry calculations from human whole-body scan data using the adult and adolescent phantoms to provide dose estimates in both populations for ^11^C-UCB-J, a marker of synaptic density.

During dosimetry analysis, it is useful to confirm that the total decay-corrected activity in the PET images remains constant across all frames and accounts for nearly 100% of the injected activity (assuming no voiding). Initially, our PET reconstructions were performed without point spread function (PSF) modeling and using relative scatter correction, in which the computed scatter sinogram was scaled to the emission data outside the subject [19]. However, these data exhibited a large drop in total activity by 26 ± 5% of injected activity from 60 to 120 min (~ 3 half-lives for ^11^C). We first evaluated whether this error was due to the scatter correction method and performed scatter correction with absolute scatter scaling [20], where scale factors are determined without using emission data outside the subject. While this removed the large drop in activity at 60 min, there remained slower reductions of 28 ± 15% of injected activity at later time points, at ~ 100–120 min. After the inclusion of PSF in the reconstruction, we found that the total activity remained constant throughout the scan at 96 ± 4% of the injected activity, consistent with a small loss of counts from the portion of the legs which were out of the scanner FOV. It has been demonstrated previously that PSF implementation is important for avoiding low-count bias in PET reconstructions [21].

It should be noted that the dose estimates in this study do not assume any urinary bladder voiding. Even though the dose-limiting organ is the gallbladder, urinary bladder voiding may decrease the ED. In the current study, one participant voided their bladder at 65 min post injection. In order to correct the bladder AUC, we examined the total counts in the image volume before and after voiding time (*t*_v_) and estimated that 15% of the total decay-corrected activity (*A*_v_) was lost due to voiding. To calculate the missing AUC_bladder_, the AUC of *A*_v_ from *t*_v_ to infinity was calculated. This AUC correction increased the urinary bladder residence time in this subject from 0.0129 to 0.0229 h. The mean ED for all subjects was then calculated using the corrected bladder residence time. The bladder voiding in this individual allowed us to examine the effect of bladder voiding on ED, which decreased by only 5%, from 0.00635 to 0.00602 mSv/MBq. Hence, bladder voiding does not appear to have a significant effect and is not necessary to be considered in dosimetry calculations for ^11^C-UCB-J.

The brain residence time for ^11^C-UCB-J in NHP was essentially the same as that in humans (− 1% difference, Table 3), indicating similar uptake and clearance of ^11^C-UCB-J in the brain, our main organ of interest. Differences in the plasma levels of parent and radiolabeled metabolites between species also need to be considered with respect to the delivery of the tracer to different organs. The parent fraction of ^11^C-UCB-J was 39% at 30 min and 24% at 90 min in NHP [22], compared with 28% at 30 min and 25% at 90 min in human [3], suggesting more rapid tracer metabolism in humans. Interestingly, the residence times in the liver differed only by 1% between NHP and human (Fig. 3, Table 3). Major differences in residence times between NHP and human were seen in the lower intestines, pancreas, stomach, gallbladder, and bladder, which were 66–96% shorter in NHP (Fig. 3, Table 3). The shorter residence times in digestion-related organs (lower intestine, pancreas, stomach, and gallbladder) are likely due to decreased gastrointestinal motility when using anesthesia (ketamine/isoflurane) and the anticholinergic, glycopyrrolate, during the NHP PET scans. Glycopyrrolate has been previously reported to dramatically decrease gastric motility in dogs [23]. Isoflurane and ketamine have also been demonstrated to decrease gastric motility [24, 25]. The large differences in radiation dose estimates between NHP and humans in the lower intestines, gallbladder, and urinary bladder, the dose-limiting organs in our current study, reflect the caution that must be taken when extrapolating dosimetry values from preclinical models, such as NHP, to human. In fact, the average ED estimated from our current human study (7.6 μSv/MBq) was 2.2 times higher than that calculated from the NHP study (3.4 μSv/MBq) [22].

A previous review of radiation dosimetry for ^11^C-labeled radioligands demonstrated a mean ED of 5.9 μSv/MBq over 42 studies [26]. Seven of these studies had the gallbladder as the dose-limiting organ with mean ED of 4.9 μSv/MBq. Our adult mean effective dose was 7.6 μSv/MBq, which is in the range of 31 of the 32 reported human studies (3.2–7.8 μSv/MBq), with ^11^C-WAY100635 being an exception with an ED of 14.1 μSv/MBq. Our adolescent mean effective dose was higher than that estimated in adults (8.8 μSv/MBq), since the activity is deposited into smaller organ volumes in adolescents. In calculating adolescent dosimetry, normalization of the adult TACs to the adult female/15-year-old (55 kg) adolescent phantom is performed. With the assumption that residence times are similar in the adult females and adolescents, the uncertainty for using the adolescent OLINDA phantoms should be minimal. However, it is difficult to extrapolate to younger ages prior to adolescence due to much lower weights and larger differences in organ and total body size as well as differences in biodistribution that will affect residence time estimates. Even so, the maximum radiation exposure from a single 188 MBq (5.1 mCi) administration of ^11^C-UCB-J is equivalent to 1.7 mSv (0.17 rem). Given that the maximum allowed annual exposure for a research subject under 18 years of age is 1.5 rem (21 CFR 361.1), multiple injections can be performed in healthy adolescents per year.

Only two previous studies specifically examined adolescent dosimetry with ^11^C-labeled radioligands [27, 28]. In order to provide context for our adolescent dosimetry results and since ED was not reported in the other studies, we compared effective dose equivalent (EDE) to the two previous studies. ^11^C-UCB-J had a higher EDE (8.8 μSv/MBq) than both ^11^C-methionine (6.6 μSv/MBq) [27] and ^11^C-PK11195 (5.3 μSv/MBq) [28]. For ^11^C-UCB-J, three of the highest uptake organs (Fig. 1; brain, bladder, and liver) had residence times that were on average ~ 5 times longer than those of ^11^C-PK11195. Tracers with focally concentrating distributions such as ^11^C-UCB-J will have higher effective doses than those that are more diffusely distributed across multiple organs. The rank order of increasing EDE among the three ^11^C-labeled radioligands in adolescent dosimetry studies (^11^C-UCB-J > ^11^C-methionine > ^11^C-PK11195) supports the pattern of their increasingly focal uptake in specific organs.

Given that the onset of schizophrenia, ASD, and other disorders often occur during adolescence, and in order to fully utilize ^11^C-UCB-J PET imaging in adolescents to understand the etiology of these diseases, radiation doses will have to be kept low. Schizophrenia has been proposed as an ideal disease for studying synaptic pruning [10], whose onset is usually in late adolescence [29], so adolescent PET imaging of those at risk for schizophrenia may allow for a deeper understanding of synaptic changes in vivo during this critical time period. In ASD, several risk genes have been identified as regulators of synaptic plasticity. These risk genes are responsible for regulating synaptic proteins, cellular receptors, and other molecules required for the regulation of neuronal synapses [13]. Thus, the ability to measure synaptic density in vivo in adolescents with ASD is also vital to understanding the role of synaptic pruning in the development of ASD.

Currently, our single-study dose limit for adolescents is 188 MBq (5.1 mCi). Due to its high brain uptake, ^11^C-UCB-J provided extremely high image quality and low test-retest variability of distribution volume estimates with a mean injected activity of ~ 15 mCi in adults (see [5] for example). A dose reduction strategy using 1/3 of the adult dose to reach the adolescent dose limit would increase noise by $\sqrt{3}$, which would still provide images with low statistical noise. Given that adolescent and adult body weights differ (e.g., 55 kg vs. 70 kg), bioavailability of tracer in plasma, and thus delivery to the brain, could be expected to be higher in adolescents. We examined bioavailability differences in a cohort of adults (20–39 years old, weights from 52.5 to 107.9 kg, *n* = 34) who underwent PET imaging with ^11^C-UCB-J by calculating the AUC of the arterial input functions, normalized to injected activity and corrected for plasma-free fraction. We found a negative correlation of normalized plasma AUC with increasing weight (*R* = − 0.48, *p* = 0.004). Thus, we expect higher bioavailability of tracer for equal injected activity in adolescents with lower weight resulting in higher activity levels in the brain, providing better statistical quality. In addition, if adolescent brains are still in the process of synaptic pruning, there may be higher SV2A levels compared to adults, which would lead to higher distribution volumes in adolescents and higher statistical counts in the brain [30]. Studies in adolescents with ^11^C-UCB-J will be needed to evaluate these hypotheses.

## Conclusions

We have provided dosimetry estimates for ^11^C-UCB-J for the study of synaptic density in adult and adolescent populations. ^11^C-UCB-J shows radiation dose estimates in the range of other ^11^C-labeled tracers. These limits should permit research studies in adolescents with ^11^C-UCB-J.

## Data Availability

The datasets used and/or analyzed during the current study are available from the corresponding author on reasonable request.

## Abbreviations

^11^C: Carbon-11
AUC: Area under the curve
ASD: Autism spectrum disorder
CT: Computed tomography
ED: Effective dose
EDE: Effective dose equivalent
FOV: Field of view
NHP: Non-human primates
PET: Positron emission tomography
PSF: Point spread function
ROIs: Regions of interest
SUV: Standardized uptake values
SV2A: Synaptic vesicle protein 2A
TAC: Time-activity curves
